# Microarray estimation of genomic inter-strain variability in the genus *Ectocarpus *(Phaeophyceae)

**DOI:** 10.1186/1471-2199-12-2

**Published:** 2011-01-13

**Authors:** Simon M Dittami, Caroline Proux, Sylvie Rousvoal, Akira F Peters, J Mark Cock, Jean-Yves Coppée, Catherine Boyen, Thierry Tonon

**Affiliations:** 1 UPMC Univ Paris 6, UMR 7139 Marine Plants and Biomolecules, Station Biologique, 29680 Roscoff, France; 2 CNRS, UMR 7139 Marine Plants and Biomolecules, Station Biologique, 29680 Roscoff, France; 3 Current Address: Department of Biology, University of Oslo, P.O. Box 1066 Blindern, N-0316 Oslo, Norway; 4 Institut Pasteur, Plate-Forme 2- Puces à ADN, 25 rue du docteur Roux, 75724 Paris Cedex 15, France; 5 BEZHIN ROSKO, 40 rue des pêcheurs, 29250 Santec, France; 6 MBA Laboratory, Citadel Hill, Plymouth PL1 2PB, UK

## Abstract

**Background**

Brown algae of the genus *Ectocarpus *exhibit high levels of genetic diversity and variability in morphological and physiological characteristics. With the establishment of *E. siliculosus *as a model and the availability of a complete genome sequence, it is now of interest to analyze variability among different species, ecotypes, and strains of the genus *Ectocarpus *both at the genome and the transcriptome level.

**Results**

We used an *E. siliculosus *gene expression microarray based on EST sequences from the genome-sequenced strain (reference strain) to carry out comparative genome hybridizations for five *Ectocarpus *strains: four *E. siliculosus *isolates (the male genome strain, a female strain used for outcrosses with the genome strain, a strain isolated from freshwater, and a highly copper-tolerant strain), as well as one strain of the sister species *E. fasciculatus*. Our results revealed significant genomic differences between ecotypes of the same species, and enable the selection of conserved probes for future microarray experiments with these strains. In the two closely related strains (a male and a female strain used for crosses), genomic differences were also detected, but concentrated in two smaller genomic regions, one of which corresponds to a viral insertion site.

**Conclusion**

The high variability between strains supports the concept of *E. siliculosus *as a complex of cryptic species. Moreover, our data suggest that several parts of the *Ectocarpus *genome may have evolved at different rates: high variability was detected particularly in transposable elements and fucoxanthin chlorophyll a/c binding proteins.

## Background

Brown algae are multicellular and almost exclusively marine organisms, which live along the coastlines of all continents. They are economically important [1] as a food product mainly in Asian countries, as animal food or fertilizer due to their high mineral and trace element contents, and as a source of polysaccharides such as alginates. More recently, additional uses, *e.g. *as a resource for drug development, as a biofuel resource, or as nutrient- and heavy metal uptake systems, have also been explored (see [2] for a review). Brown algae are ecologically significant as they form the dominant vegetation in the intertidal and subtidal zone of rocky shores; large species, such as giant kelps, provide habitats for many other organisms [2]. Being part of the heterokont lineage within the chromalveolate kingdom, brown algae have evolved independently from other multicellular eukaryotes, including land plants and red and green algae [3]. In spite of their importance, there are still many gaps in our knowledge about brown algae, such as the mechanisms involved in their development, their complex life cycles [4], and their responses to stress.

Among brown algae, *Ectocarpus siliculosus *has a long history of research [5], and was chosen as a genetic model [6] due to its small genome and its short life cycle. Its genome has recently been sequenced and annotated, and is the first available for any seaweed [7]. Until recently, it was generally accepted that the genus *Ectocarpus *included only two species, *E. siliculosus *and *E. fasciculatus*. The cosmopolitan *E. siliculosus, *however, shows a particularly high level of genetic diversity and probably contains several cryptic species; one of which has been taxonomically re-instated as *E. crouaniorum *[8-10].

In addition to this genetic diversity, *Ectocarpus *also exhibits a considerable degree of physiological plasticity, and some strains have been isolated from quite extreme physiological conditions, such as freshwater [11,12] and a site that was severely polluted with heavy metals [13]. Such ecotypes constitute a valuable resource for the study of adaptation to different environments, as demonstrated by numerous reports for terrestrial plants, comparing *e.g. Arabidopsis thaliana *and the closely related halophyte *Thellungiella salsuginea *(reviewed in [14]). In *Ectocarpus *a similar comparison of two strains has been performed on a proteomic level, highlighting for instance the importance of a photosystem II Mn-stabilizing protein and of a fucoxanthin chlorophyll a/c binding protein (FCP) during the adaptation to high levels of copper [13].

Microarray experiments could provide valuable insights into the biology of different ecotypes as well as into their specific adaptations, as they allow transcript abundances to be assayed for a large number of genes at a comparatively low cost. Currently, an expression array based on the genome-sequenced strain of *E. siliculosus *is available, which comprises 68,270 probes for 17,119 sequences, including 8,165 contigs and 8,874 singletons from several expressed sequence tag (EST) libraries [15]. However, considering the present uncertainty with respect to the presence of cryptic species within *E. siliculosus *and physiological differences between the strains, caution needs to be taken when using this array for other strains [16]: cross-hybridization, alternative splicing, and sequence divergence may significantly decrease the accuracy of such experiments.

Comparative genome hybridization (CGH) experiments, using expression arrays and genomic DNA (gDNA), have been used as a means of assessing the suitability of microarrays for cross-strain and/or cross-species hybridizations. This was first demonstrated by Ranz et al. [17] for two closely related species of *Drosophila*, and has been successfully applied in land plants [18,19]. The results from such CGH experiments can be used to mask probes with high inter-strain and inter-species variability, thus increasing the accuracy of expression analyses carried out with alternative strains or species. Moreover, in spite of the limitations imposed by the use of gene expression arrays, cross-species hybridizations may also yield information on rapidly evolving or highly conserved gene sets. For example, in a recent analysis of two related species of soybean, the highest degree of conservation was observed for genes involved in basic metabolic processes such as photosynthesis, while a high degree of variability was observed for signal transduction genes such as transcription factors [20].

In this study, we used a similar approach. CGH experiments were performed with five different strains of *Ectocarpus *and three objectives in mind: 1) to estimate the genomic variability between strains; 2) to facilitate future cross-strain gene expression experiments by enabling the masking of divergent probes; and 3) to identify possible rapidly evolving gene families and/or genomic regions.

## Results and Discussion

### Selection of strains

Five *Ectocarpus *strains from different origins (Table 1) were selected based both on their phenotypic characteristivcs and on their classification within the taxonomic clades defined by Stache-Crain *et al. *[8]. In our study, the species name *E. siliculosus *is used to refer collectively to clades 1-4 of this phylogeny.

**Table 1:** Accession numbers, origin, and description of strains used in our experiments

Strain number in this paper	Strain characteristics	Number in *Ectocarpus *strain collection at SBR	CCAP accession	Origin	Species	*Ectocarpus *clade	*ITS1 sequence accession*
1	Genome-sequenced strain, male partheno-sporophyte from fully marine environment	Ec 32	1310/4	San Juan de Marcona, Peru	*E. siliculosus*	1c	AJ550048
2	Female partheno-sporophyte, sexually compatible with strain 1, from fully marine environment (subtidal 3 m)	Ec 568	1310/334	Arica, Chile	*E. siliculosus*	1c	FN564446
3	Freshwater strain, unknown sex or life-history stage	Ec 371	1310/196	Hopkins River Falls, Victoria, Australia	*E. siliculosus*	2d	GQ351370
4	Copper-tolerant strain, unknown sex or life-history stage	Ec 524	1310/333	Palito La Boca, Chañaral, Chile	*E. siliculosus*	1a	FN564444
5	Outgroup, from fully marine environment (upper subtidal, epiphytic on *Himanthalia*), unknown sex or life-history stage	Ec 395	-	Roscoff, France	*E. fasciculatus*	5b	FN564441

*Ectocarpus *clade numbers [54,9,10] correspond to a previous phylogenetic analysis [8]. SBR = Station Biologique de Roscoff, CCAP = Culture Collection of Algae and Protozoa, Dunstaffnage, Scotland http://www.ccap.ac.uk/.

**Figure 1:**
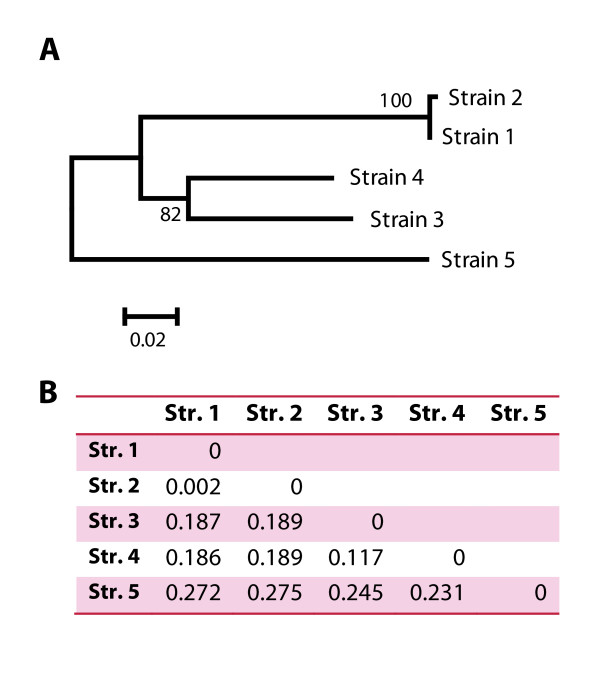
**Phylogenetic relationships between the five *Ectocarpus *strains examined in this study (Str. 1-5) inferred from the ITS1 region**. A) Neighbor-joining tree (BIONJ, default parameters) of the ITS regions of strain 1-5; 100 bootstrap replicates. B) Corresponding distance matrix for the tree in panel A.

Strain 1 is the genome-sequenced strain of *E. siliculosus*. ESTs produced for this strain were used for the design of the gene expression array. This strain falls into clade 1c of the Stache-Crain et al. [8] phylogeny, and served as a reference strain for all hybridizations. Strain 2 falls into the same clade [9] (Table 1), and is known to be cross-fertile with strain 1, but exhibits a high degree of genetic polymorphism. Strains 1 and 2 were used to construct the recently published genetic map for *Ectocarpus *[21]. Strain 3 was chosen as it is the only well documented isolate from freshwater [11,12]. Its internal transcribed spacer (ITS) 1 region, which was sequenced in this study, revealed that it falls into clade 2d. Strain 4, which belongs to clade 1a, was of particular interest due to its high tolerance to copper and also because of the available proteomic data [13]. Finally, strain 5 belongs to a different species (*E. fasciculatus*), and was chosen as an outgroup to assist the interpretation of the degrees of variance observed within the different *E. siliculosus *strains. The relative genetic distances between the examined strains, based on an alignment of the ITS1 region, are displayed in Figure 1.

### Reliability of the CGH experiments

In order to assess the reproducibility of our CGH experiments, a reference-reference hybridization was carried out with gDNA from two independent cultures of strain 1 (labeled with Cy3 and Cy5 respectively). The results of this experiment demonstrated that only 166 of the 68,270 probes (0.24%) exhibited log2-differences in signal intensity > 1 (*i.e. *> 2-fold change). A more detailed examination revealed that 90 of these 166 probes (54%) were not associated to a genomic supercontig (Sctg). Overall this was the case for 2,676 probes (3.9%, see Methods for additional details), indicating that a part of these sequences might correspond to contamination in the ESTs and/or to low complexity regions that are difficult to sequence.

**Figure 2:**
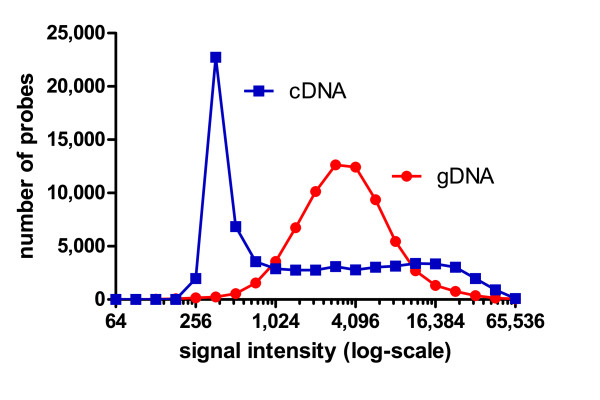
**Comparison of microarray experiments performed by hybridization of gDNA and cDNA**. The gDNA curve corresponds to the distribution of the Cy3-channel signals from the reference-reference experiment, while the cDNA curve represents the first control sample of a previous gene expression experiment carried out with the same strain and the same array under similar hybridization conditions [15]. In cDNA experiments, many targets are present at low copy number (low signal) and a few sequences are present in high copy number (high signal). In contrast, in gDNA experiments, most targets are present at the same copy number, resulting in a peak at medium intensity.

The reference-reference experiment therefore demonstrated a high degree of technical reproducibility in microarray experiments employing gDNA. One reason for this can be found in the distribution of absolute signal intensities obtained with gDNA, compared to cDNA (Figure 2). Genomic DNA-based CGH experiments result in signal intensity distributions with a maximum at medium signal intensities and thus high signal to noise ratios, because all genes are present in similar copy numbers. In contrast, cDNA or RNA experiments need to accommodate large differences in transcript abundance, resulting in many probes giving low signals and overall lower signal to noise ratios. In the light of these findings, and as the nuclear genome within the same strain may be assumed to be constant, all CGH experiments were only carried out with a single replicate. Changes in the content of organellar DNA could theoretically also be detected using our experimental setup. However, this would require testing biological replicates as the number of organelles and/or their DNA content may be subject to variations according to the conditions of the culture [22-24]. These changes were therefore not examined in this study.

### Marked genetic differences support the presence of cryptic species

**Figure 3:**
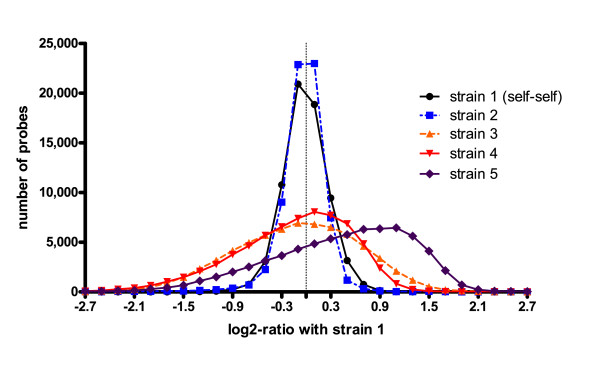
**Distribution of log2-ratios (sample strain/reference strain 1) in the examined strains of *Ectocarpus***. "Strain 1" designates a reference-reference hybridization of two independent samples of strain 1. The apparent shift of the curve for strain 5 towards positive values is likely to be an artefact caused by the normalization procedure. Probe frequencies were calculated in intervals of 0.2.

CGH analysis of the different strains indicated that strains 1 and 2, which are known to be fully compatible [9,21], have very similar genome sequences: the standard deviation of the log2-ratios from the array comparison for these strains was 0.3 (see Figure 3 for a distribution of log2-ratios), which was the same as that obtained for the reference-reference hybridization using two independent samples of strain 1, and close to values obtained for similar experiments in bacteria [25].

In comparison, for the freshwater- and copper-tolerant strains (strains 3 and 4), standard deviations, compared to the reference strain, were 0.8 and 0.7 respectively. These values were close to the value obtained for the outgroup strain (*E. fasciculatus*, strain 5), which was 0.9. These data agree well the phylogenetic tree of the examined strains (Figure 1), supporting the idea that *E. siliculosus *may be a complex of several (cryptic) species [8-10].

### Selection of conserved probes for future microarray experiments

In spite of the marked genetic differences between strains, analysis of the DNA hybridization data showed that the microarray can still be exploited to analyze gene expression in all the strains tested except for strain 5 (see below), provided only conserved probes are selected for the analysis [19]. If a very stringent threshold for masking probes in cross strain experiments was chosen, *e.g. *0.5 (1.4-fold change in signal intensity), a number of probes could be retained from microarray experiments: 64,608 (95%), 32,501 (48%), and 36,336 (53%) for strains 2, 3, and 4, respectively. Moreover, because each sequence is represented by four probes, expression profiles may be obtained for 16,845 (98%, strain 2), 14,554 (85%, strain 3), and 15,078 (88%, strain 4) sequences, respectively. In many cases, *i.e. *in experiments that do not rely on direct inter-strain comparisons but rather on comparison of the same strain submitted to different treatments, a less stringent cut-off such as an absolute log2-ratio of 1 may be more appropriate, and would allow even more probes to be retained.

For strain 5, our current analysis does not provide any reliable selection criteria for conserved probes, as Figure 3 indicates that a bias might have been introduced during the normalization procedure. Unless specific probes are used for normalization, most normalization algorithms assume that the majority of probes yield similar signals for both of the examined samples. Although we used the popLowess algorithm [26], which has been designed to be less sensitive to copy number imbalances (or changes in sequence), we observed a high number of probes that exhibit a log2-ratio of 1.1 (Figure 3) for strain 5. The maximum number of probes would be expected at a log2-ratio of 0, as for the other strains, and a shift towards positive values suggests that the number of divergent probes was too high for the algorithm to function correctly. This strain was therefore excluded from further analyses.

To facilitate the selection of probes for strains 2 to 4, we created a Java application, which can be used to remove a list of probes from raw pair files, prior to normalization using the NimbleScan software (Additional file 1). Along with this program, we also provide a list of all probes with log2-changes greater than 0.5 and greater than 1. In addition, this program could also be applied to our data to pre-select probes based on their absolute signal intensity rather than the similarity between test- and reference strain. This approach has been suggested to decrease noise in RNA-based cross-species hybridizations [20,27], but was not further explored here, as unlike in typical gene expression experiments, almost all probes produced medium to high intensity signals (Figure 2).

### Putative deletions/duplications were detected mainly in strain 2

To determine whether the divergent probes were distributed randomly throughout the genome, the normalized log2-ratios of strains 1-4 were analyzed at two levels: at the regional level, in order to determine highly variable genomic regions as well as duplications or deletions, and at the gene or EST level, to determine if particular functional groups of genes exhibited higher differences than others.

**Figure 4:**
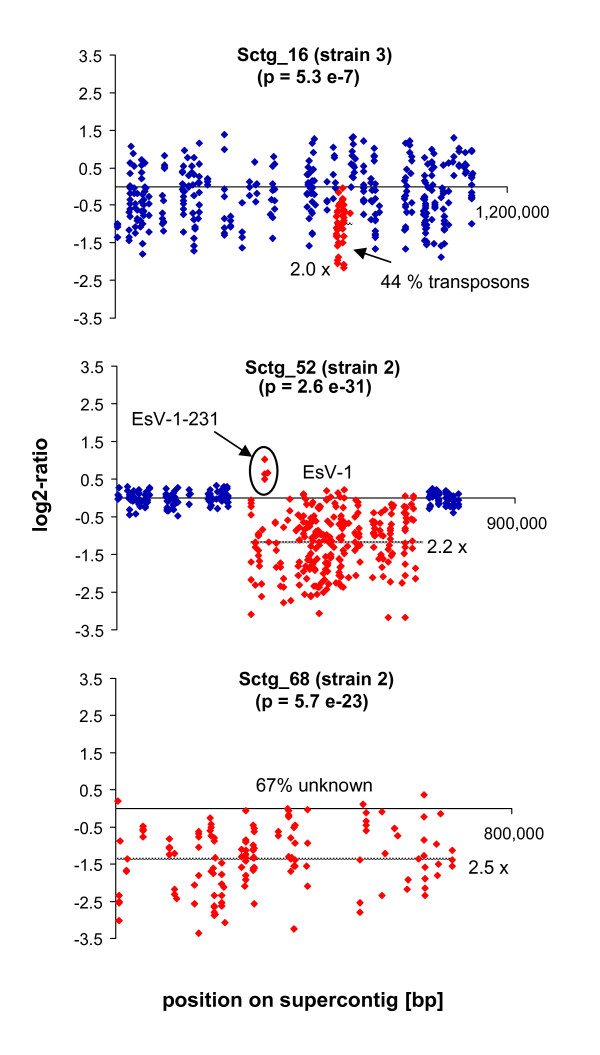
**Genomic regions with significant differences in signal intensity in the examined *Ectocarpus *strains**. The graphs display the log2-ratio between sample and reference strain (1) for each probe. A log2-ratio of 0 means that there was no difference between the examined strains. Red dots represent probes in regions with significant differences between the test and the reference strain (1), blue dots the surrounding probes (if present). The grey dotted line indicates the mean log2-ratio over the highlighted area; the corresponding fold-change is given followed by an "x". *E. fasciculatus *(strain 5) was not included in this analysis (Sctg = supercontig, EsV = *Ectocarpus siliculosus *virus).

For the first (regional) analysis, microarray probes were positioned on the various genomic supercontigs and sets of 30 probes were screened using a sliding window approach (see Methods). Three areas with markedly different hybridization patterns were detected (Figure 4). Each region was then examined using quantitative PCR (Table 2). One of the three differences was found in strain 3, where a small region on Sctg_16 containing mainly transposable elements (TEs), had significantly lower signals compared to the reference strain (2-fold in the CGH experiment, 1.2-fold in the quantitative PCR validation), and will be discussed below. The two other regions were both found to differ between strains 1 and 2, which are the genetically closest strains. One concerned the *E. siliculosus *virus 1 (EsV-1), and the second a rather small genomic Sctg, both of which will be discussed in the following section.

**Table 2:** Quantitative PCR validation of the CGH experiments

Gene/region	forward primer	reverse primer	amplification efficiency (%)	reference strain	experimental strain	ratio (mean ± SD, n = 3)	ratio for region (CGH)
R26S (ref. gene)	GCTAGGCTTGCGTTTGTGTG	GGCGAGACAGAAAGATTCCG	108	strain 1	strain 2/3	-	-
Dynein (ref. gene)	GGAACAAAGCATGGTGACAACA	CGCGTGCCTATCCAAGCT	97	strain 1	strain 2/3	-	-

Sctg_16	GCGTGCGTGCTTGGAAGG	TTCGGCTGCTGAGAGTGGAG	96	strain 1	strain 3	0.9 ± 0.04	0.5
Sctg_16	CAACCGCTCTCCACCATTCAG	GACGCCTTCACAGTATCACACC	96	strain 1	strain 3	0.7 ± 0.02	
Sctg_16	AACGATAGAGCGAGACGAGAGAG	GGAAGCAGATGGACACGAGTAAC	93	strain 1	strain 3	0.8 ± 0.03	

Sctg_68	CTCCTATCGCCCTGTGGTCTC	ACTGCCTCTATGGTCCGTCTTG	100	strain 1	strain 2	1.0 ± 0.1	0.4
Sctg_68	GTGAGAGAAACAACAGAGCAATACAG	ATGGAACCGCAGACAACAAGC	102	strain 1	strain 2	0.6 ± 0.2	
Sctg_68	TCCGACCTGACGAGCATTGG	CAGTGTGCGGTGCGAACG	103	strain 1	strain 2	n/a*	
Sctg_68	AAACACCTCCCAACCAACCAATC	AACGCAACGAGCAACCTTCC	100	strain 1	strain 2	n/a*	

EsV-1	TAAGTTGATATTAGTGACAGTAGCAGGAG	GCCACGGAGGACGGAGATAC	101	strain 1	strain 2	n/a*	0.5
EsV-1	ACCACGATGCCTGTCTCCTTAC	TCCTCAGCCGCCAGAATACG	95	strain 1	strain 2	n/a*	
EsV-1	CTCCTCCGTAACCGTTGACATTG	CCGACCAGTAAACCCGTAAACC	101	strain 1	strain 2	n/a*	

* no amplification in experimental strain, or difference > 10 cycles

Primers and validation results for each tested genomic region (EsV-1 = region on Sctg_52 containing the *E. siliculous *virus 1 genes, ref. gene = reference gene). The amplification efficiency was calculated from the standard curve for strain 1.

### Differences with respect to a viral integration site and to a region of unknown function between strains 1 and 2

In strain 1, a large DNA virus closely related to EsV-1 [28] was identified in genomic Sctg_52. In spite of the presence of this virus in the genome, symptoms of viral infection have not been observed in this strain, and transcriptomic data suggested that the viral genes are not transcribed [7]. As strains 3 and 4 showed similar signal intensities in this region compared to the reference strain, both strains may also contain the viral genome, although, as with the reference strain, production of viral particles was not observed.

In strain 2, the region of the viral insertion on Sctg_52 exhibited 2.2-fold lower signal intensities compared to strain 1 (Figure 4). Nevertheless, for several genes of this Sctg, the log2-ratio between the two strains reached zero, and even positive values in one case (viral gene EsV-1-231, Figure 4). As the viral genome is present in a single copy in the reference strain, this difference could be due either to the absence of viral sequences within the genome of strain 2, in which case the remaining signals for strain 2 could be explained either by non-specific binding, or by the presence of highly divergent EsV-1-like sequences, such as a degenerated version of EsV-1. In cultures of strain 2, we have not observed any symptoms of viral infection.

An alternative explanation can be provided by an observation made in a previous study: Müller *et al. *[29] detected amplification of a viral gene in a population of *Ectocarpus sp. *at different annealing temperatures depending on the individual, suggesting the presence of several distinct, but genetically similar, viruses within the same population. The hypothesis that strain 2 contains such a related *E. siliculosus *virus integrated into its genome would agree with the profiles observed in this study. Further information about the viral genes potentially present in strain 2, including their insertion sites, might provide clues as to which common features could be responsible for the silencing of viral gene expression.

The second region exhibiting significant differences between strain 1 and strain 2 was a small supercontig (Sctg_68). Just as for the EsV-1 region, signals were significantly lower in strain 2 (2.5-fold on average), and in the quantitative PCR analysis two of four primer pairs amplified only in strain 1, while two others indicated no or only a 1.7-fold decrease (factor 0.6, Table 2) in strain 2. Again, these differences could be due to two reasons: deletion(s), or very high variability of this region in strain 2. The first hypothesis seems unlikely because of the wide range of differences in signal intensities on Sctg_68 (log2-ratios from -3.4 to 0.4), comprising several probes with ratios close to 0. Furthermore two of the four primer pairs also yielded amplicons in strain 2. Regarding the second hypothesis based on low sequence identity between the strains, sex related differences could provide a possible explanation and work is currently being carried out to test this hypothesis (Coelho & Cock, personal communication). Sctg_68 is predicted to encode 21 proteins, 14 of which are (conserved) hypothetical proteins with unknown functions.

### Functional analysis of highly conserved and highly variable genes

To identify functional groups of genes that were subject to particularly high conservation or variation, we examined each of the contigs and singletons used for the design of the array. Contigs and singletons were defined as "conserved" if none of the four probes associated with each sequence exhibited an absolute log2-ratio with the reference strain > 1, and as "variable" if two or more probes exhibited an absolute log2-ratio with the reference strain > 1. We then searched for enrichment of GO terms among the sequences classified as variable for strains 2-4, as well as among the sequences classified as "conserved" in all of these strains.

One of the problems with this sort of analysis is that probes located within the untranslated region (UTR) are usually less conserved than probes located in the coding sequence (CDS). In our dataset the overall proportion of probes located within the UTR of a gene was 62% (42,073/68,270). However, when considering only the most variable probes (absolute log2-ratio > 1) this percentage increased to 67% (688/1,012), 73% (9,585/13,141) and 84% (8,764/10,369) in strains 2, 3, and 4 respectively. This phenomenon will be termed UTR bias hereafter, and could potentially lead to the identification of functional groups of genes as highly variable or highly conserved, based on the percentage of probes that have been designed in the UTRs for this group. Therefore, in the following section, we assess the percentage of CDS or UTR probes for each functional group identified, and perform comparisons using both the entire data set as well as only the UTR probes as reference where necessary.

### A set of 7,497 sequences were conserved in all the *E. siliculosus *strains analyzed

**Figure 5:**
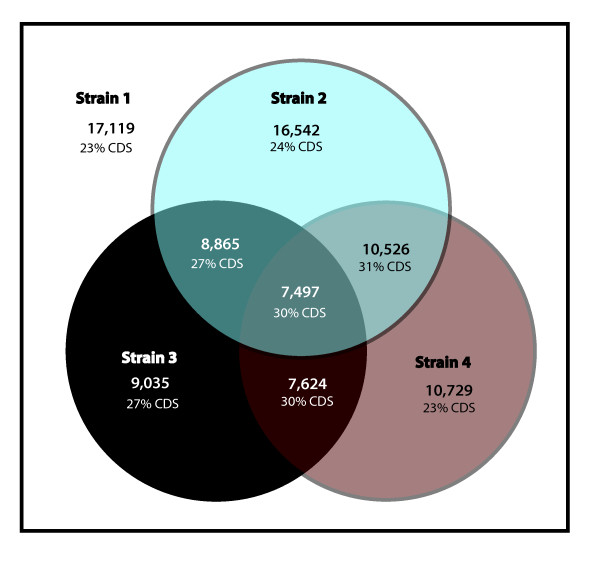
**Venn diagram indicating the number of conserved sequences in tested strains of *E. siliculosus***. A sequence was considered to be conserved when all four probes corresponding to this sequence exhibited an absolute log2-ratio with the control < 1. The first number indicates the total count of ESTs derived sequences (singletons and contigs) conserved between the strains, and the percentage of these sequences represented only by probes in the CDS region is given below. The number in the center of the graph, for example, indicates that 7,497 sequences are conserved between strain 1, 2, 3, and 4, while the number in the blue circle above shows that 16,542 sequences were conserved between strain 1 and strain 2. Please note that strain 1 is the basis of all comparisons, as only genes that are present in this strain are represented on the array. Strain 5 was not included in this analysis due to the bias introduced by the normalization procedure.

In accordance with our estimation of the overall genetic differences between the examined strains, we found that in strain 2 97% of all sequences were considered conserved with respect to strain 1 (absolute log2-ratio < 1 for all four probes), while in strains 3 and 4 this was only the case for 53% and 63% of the sequences, respectively (Figure 5). These findings are in agreement with the ITS tree and the corresponding genetic distances reported in Figure 1. Furthermore, we identified a set of 7,479 (44%) core sequences, which were considered conserved in all four examined strains of *E. siliculosus*. An automatic analysis of these sequences highlighted only one GO (Gene Ontology) category (FDR < 0.05): "Structural constituent of ribosomes". In contrast to this, a similar study conducted between two soybean species [20] identified numerous GO terms, including some related to photosynthesis and transporters. The differences between these two studies may, however, be related to the respective methodological approaches. While Yang et al. [20] examined absolute signals derived from hybridization of cRNA, we examined the relative change in signal from gDNA hybridization and thus eliminated any possible bias introduced by differences in gene expression levels. An assessment of the effects of the UTR bias on the results obtained for sequences annotated as structural constituents of the ribosome in our study revealed that only 18 (*i.e. *11%, vs. 23% in the entire dataset) contained only CDS probes (*i.e. *sequences for which all four probes are located in the CDS), and the overall percentage of CDS probes in these sequences was 40% (vs. 38% in the entire dataset). UTR bias was therefore not an issue for these sequences.

### Transposable elements and fucoxanthin-chlorophyll a/c binding proteins are among the most variable sequences

Strain 2 was compared with the reference strain 1 to identify sequences that exhibited a high degree of variability between the two strains. We found only 264 sequences that contained at least two probes with an absolute log2-ratio > 1, and an automatic search for enriched GO categories in this subset did not yield any significant results, but we identified 18 TEs (6.8% of the sequences mentioned above) that were part of the database of known *Ectocarpus *TEs [7]. In comparison, the entire dataset contains 284 known transposons (*i.e. *TEs represent 1.7% of the entire dataset).

**Figure 6:**
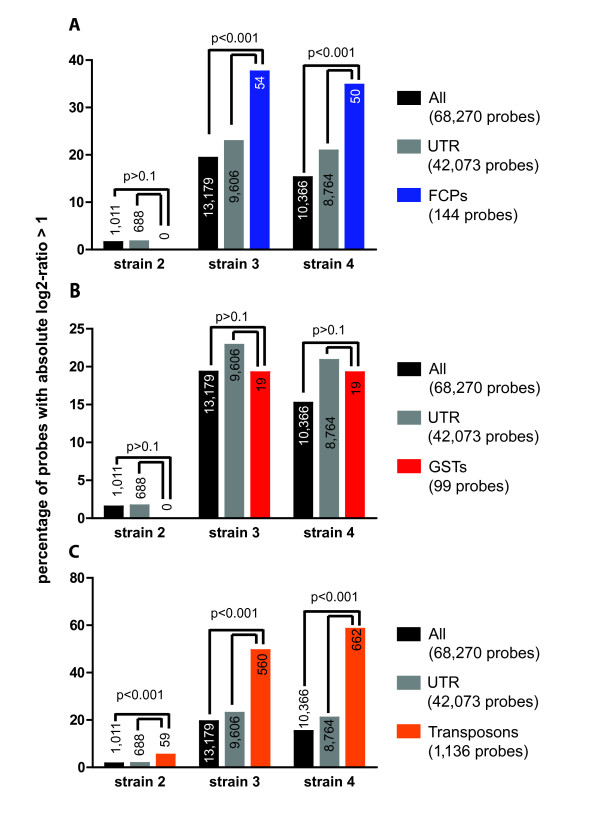
**Percentage of variable probes (*i.e. *probes exhibiting an absolute log2-ratio of test to reference strain > 1) belonging to different groups: (A, blue) fucoxanthin chlorophyll a/c binding proteins (FCPs), (B, red) glutathione S-transferases (GSTs), and (C, orange) TEs (Transposons)**. The higher the bar, the higher the degree of variability in a particular strain or group of probes. As a comparison, each graph shows also the percentage of variable probes among all probes (black) and only UTR probes (grey). P-values were calculated using a binomial test in comparison to both all probes and only UTR probes.

For strains 3 and 4, we identified 3,343 and 2,563 sequences respectively that matched our selection criteria (*i.e. *at least two probes with an absolute log2-ratio of the test strain to reference strain > 1). An automated search for enriched GO terms (FDR < 0.05) in this set of sequences yielded only one GO-category, *i.e. *chlorophyll binding, which consists mainly of fucoxanthin-chlorophyll a/c binding proteins (FCPs). We then completed the list of FCPs using a list of sequences identified by manual annotation [7] (Additional file 2). Highly variable probes were found to be significantly overrepresented also among the complete set of 144 FCP probes on the array. Although FCPs were represented by a higher proportion of UTR probes (112/144, 78%) compared to the entire dataset (62%), the over-representation of highly variable probes among FCPs was also statistically significant when comparing the FCP probes to only the UTR probes as background (Figure 6A). This confirms that UTR bias was not the primary reason for these genes being among the most variable.

In order to determine if high variability between strains was a feature common to other multigenic families, which merely remained undetected due to the lack of high quality automatic annotations for some of them, we performed the same analysis for probes corresponding to 25 manually annotated glutathione-S-transferases (GSTs; Additional file 2, [30]). Our analysis did not reveal any significant differences between GSTs and the rest of the genes (p > 0.1 Figure 6B). This shows that not all multigenic families are subject to high variability in different strains of *Ectocarpus*.

Finally, as automatic GO annotations did not include annotations for TEs, but since they were highly represented among the sequences found most variable in strain 2 (see above), they were analyzed separately. The 1,136 probes corresponding to the 284 transposons represented on the array (see Additional file 2 and Methods) were significantly overrepresented among the highly variable probes in all *E. siliculosus *strains (Figure 6C), both when the entire dataset or only the UTR probes were used as a basis for the comparison.

There may be several reasons why certain sequences are less conserved than others. Certain genes or genomic regions may be at increased risk of targeted deletions via recombination events [31,32]. Others might be essential for the adaptation to different environments, and thus subject to different selective pressures as demonstrated for rapidly evolving proteins in two species of *Arabidopsis *[33]. Although we can presently only speculate about the importance of FCPs and transposons for this latter process, both categories of sequences have been recently discussed in this context for heterokonts and other organisms.

FCPs are part of the light harvesting complex, and are thought to function primarily in the transmission of light energy to chlorophyll. Recent transcriptomic studies in *Chaetoceros *and in *Ectocarpus*, however, showed some FCPs to be transcriptionally induced in response to stress [15,34]. Other FCPs have also been shown to be differentially expressed in the gametophyte and sporophyte generations of *Ectocarpus *[35]. In the green alga *Chlamydomonas reinhardtii *[36] and in the diatom *Cyclotella meneghiniana *[37,38], FCP-related proteins have also been recently implicated in the process of non-photochemical quenching. The *Ectocarpus *genome contains a total of 53 FCPs, a multitude that may be related to the adaptation to highly variable light conditions in the intertidal and shallow subtidal zones [7,39]. Many of the *E. siliculosus *FCPs share a high degree of sequence similarity, and some are located in close proximity on the same supercontig, both observations suggesting recent gene duplications within this family. The recent expansion of the FCP family in *E. siliculosus*, as well as the evidence for high variations between different strains of *Ectocarpus *presented in this study, would agree with the hypothesis that FCPs have evolved or are evolving to serve different functions within the chloroplast, and with their potential role in the adaptation to different environments [7,39,40].

TEs are a major component of many eukaryotic genomes, and often considered as ''junk'' DNA or genomic parasites [41]. However, there may be a limited number of instances where they could confer benefits. For example, certain transposons have recently been suggested to play a role in the adaptation of *Drosophila *to temperate environments [42,43]. A study in diatoms (which are also members of the heterokont lineage) proposed that retrotransposons may promote genome rearrangements, thus possibly conferring phenotypic plasticity to an individual species, and aiding the adaptation to different environments [44]. Two important ways of controlling transposons are silencing by methylation and RNAi-like mechanisms [41]. Our findings that TEs were among the most variable components of the *Ectocarpus *genome, and that even very closely related strains (strains 1 and 2) differed with respect to these sequences, are in agreement with the observation that TEs in *Ectocarpus *are both highly expressed and are not methylated [7].

Neither in the case of transposons nor in the case of FCPs does our study present any proof of a direct relationship to the adaptation to different or extreme environments. It does, however, highlight both groups as promising subjects for future studies examining this question.

## Conclusion

This study is the first microarray based genomic comparison of different brown algal strains. It enabled the detection of significant genomic variations between different ecotypes thought to belong to the same species, supporting the hypothesis of several cryptic species within *E. siliculosus*. At the same time, it provided a set of conserved probes which can be used for future transcriptomic experiments using the microarray available for the genome-sequenced strain and analyzing three of the four examined test strains.

In addition, further analysis of the CGH results provided first indications of differences with respect to an EsV-1 insertion in the genome of one of the examined strains, highlighting a potentially interesting candidate for the study of viral diversity as well as differences in integration sites. Finally, an analysis of the most variable microarray probes demonstrated that several functional elements of the *Ectocarpus *genome were likely to evolve at different rates. Both TEs and FCPs were identified as part of the most variable elements in terms of copy number and/or sequence identity, and could be of importance in the evolution of different strains of *Ectocarpus*. Together these results pave the way for further studies to explore the biology and the adaptation of the examined ecotypes to their respective environments.

## Methods

### Algal strains and culture conditions

All strains were clonal isolates and cultivated in 10-liter plastic flasks in a culture room at 13-14°C using filtered and autoclaved natural seawater enriched according to Provasoli [45]. Although none of the examined strains were axenic, cultures were handled under axenic conditions, and bacterial contamination could not be detected using light microscopy. Cultures were irradiated by daylight-type fluorescent white light (40 μEm^-2 ^s^-1^) under a 14/10 light-dark cycle and were permanently aerated with filtered (0.22 μm) compressed air.

### DNA extraction and fragmentation, and ITS1 sequencing

Approximately 1 g (wet weight) of algal material was harvested by filtration, dried with a paper towel, and frozen in liquid nitrogen. These samples were used for DNA extraction using CsCl-gradient purification based on the protocol described by Apt *et al. *[46] with modifications as described by Le Bail *et al. *[47]. The ITS1 sequence of strain 3 was determined as described by Peters *et al. *[9], and sequences of the other strains were available from public databases. Accession numbers are provided in Table 1. For the calculation of the tree displayed in Figure 1, the BIONJ algorithm [48] was used with default parameters and bootstrapping (100 replicates). ITS sequences of strain 1-5 were aligned using MAFFT [49] and the L-INS-i strategy, and conserved bases were selected using the Gblocks server [50], allowing smaller final blocks and less strict flanking positions.

### Hybridization and scanning

The genomes of the five selected strains were analyzed by hybridizing fluorescently labeled gDNA of the five strains to an EST-based Roche NimbleGen 4-plex expression array [ArrayExpress: A-MEXP-1445]. This array represents 8,165 contigs and 8,874 singletons by four unique 60-mer probes each. [15]. The array furthermore contained probes for 231 sequences of EsV-1 [28]. A closely related virus is present as an integrated sequence in the genome of the *Ectocarpus *genome strain 1 [7]. Note that, in some cases, a gene may be represented by more than one cDNA contig/singleton. In total, the array covers about 10,600 (*i.e. *65%) of the 16,256 predicted unique genes in the genome. Strain 1 represented the reference strain. For each sample, one μg of fragmented DNA was labeled using the Roche NimbleGen Dual-Color DNA Labeling Kit (Roche NimbleGen, Madison, WI, USA) following the manufacturer-supplied CGH Analysis protocol v5.1. Reference DNA (strain 1) was labeled with Cy5 and test DNAs (strain 2-5) with Cy3. In addition, a reference-reference hybridization was carried out using two independent DNA samples from strain 1, one labeled with Cy3 and the other with Cy5. One μg of DNA was used for each labeling reaction which yielded > 4 μg of labeled DNA. Four μg of each sample were hybridized together with 4 μg of the reference DNA (strain 1), using the Roche NimbleGen Hybridization System 4 and following the standard Roche NimbleGen protocol (CGH Analysis protocol v5.1). Scanning was performed according to the same protocol using a Genepix 4200AL scanner and the Genepix pro 5.0 software (Molecular Devices, Sunnyvale, CA, USA).

### Normalization

Scanned images were imported into NimbleScan version 2.4 (Roche NimbleGen, Madison, WI, USA), and the raw signal intensity was extracted for each probe according to the Roche NimbleGen CGH Analysis user guide (available in the protocols section of our Array Express submission; see below). This protocol does not include a background subtraction step, which might lead to a slight underestimation of log2-ratios for probes with low signals. A ".pos" file (Additional file 3) for our microarray was generated by blasting each of the microarray probes against the entire *Ectocarpus *genome (EMBL accession numbers CABU01000001-CABU01013533, FN647682-FN649242, FN649726-FN649760, [7]) using the megablast algorithm [51]. Each genomic supercontig was treated as a chromosome; 2,676 probes (3.9%) could not be clearly assigned a position on the genome (homologous sequences were not found). These probes may correspond to low-quality sequences or contaminations and were assigned randomly to a "virtual" chromosome, which was later used to choose ideal parameters for the DNA copy number analysis (see below), but not considered for other analyses. Raw log2-ratios were normalized using the popLowess-algorithm version 1.0.2 [26] and R http://www.r-project.org version 2.9.1/Bioconductor version 2.3 http://www.bioconductor.org. The popLowess algorithm selects a subset (a population) of probes with very similar signals and uses this subset to normalize the entire dataset, thus making the algorithm less sensitive to copy number imbalances (or changes in sequence). The following parameters were used: significance threshold for accepting change points = 0.05, smoother span = 1/3, 4 iterations, and δ = 0.1.

### Statistical and functional analysis

Normalized log2-ratios of strains 1-4 were analyzed at two levels: at the regional level by examining sets of 30 probes using a sliding window approach, and at the gene or EST (singletons and contigs) level. For the analysis at the regional level, normalized expression values were imported into the Partek Genome Suite software version 6 (Partek Inc., St. Louis, MO, USA), which was used for scanning for copy number alterations using circular binary segmentation (CBS, [52]). This method detects regions with potential duplications and deletions in the genome, and assigns them a p-value. Please note that these p-values, unlike those from the qPCR validation, are merely based on the signal intensities of different probes within one biological replicate. For our analysis, only segments with at least 30 probes and a mean log2-ratio greater than 1 or less than -1 were considered, because these settings yielded no false positives on the "virtual" chromosome, while still allowing to detect relatively short deletions or duplications with a minimum length of 7 to 8 genes. We chose to apply a p-value cutoff of 7.4e-7, which corresponds to a p-value of 0.05 after a Bonferroni correction for 68,240 tests (*i.e. *the maximum number of possible windows of 30 probes). Since the tested windows overlapped, the latter assumption is very conservative. However, less stringent methods would not have changed the number or identity of the identified genomic regions as the p-value of the next most significant segment was three orders of magnitude above our cutoff.

Data were also analyzed at the EST level (singletons and contigs represented on the array). We selected all sequences with at least 2 of the 4 probes showing an absolute log2-ratio between test strain and reference strain > 1, for each of the four strains, as well as sequences conserved in all strains (*i.e. *all four probes exhibited absolute log2-ratios with the reference strain < 1). Using the GO annotations generated in our previous study [15], enriched GO terms were searched for using the GOLEM software [53] and allowing a false discovery rate (FDR) of 5%. The proportion of variable probes (absolute log2-ratio > 1) in the identified groups was compared to that in all probes (UTR + CDS) as well as to that in only the UTR probes by means of a binomial test. TEs were identified by sequence homology with a database of known *E. siliculosus *transposons [7]. Only sequences with >80% sequence similarity over at least 400 bp were considered.

### Validation

Genomic regions that yielded significantly different signals between the reference and test strains were verified by real time quantitative PCR on genomic DNA of three biological replicates, as described previously [47]. Three to four fragments were amplified and quantified per region using 4 ng of gDNA as template and the primer pairs listed in Table 2. Standard curves were created to calculate the reaction efficiency for each primer pair using a dilution series of 16, 8, 4, 2, 1 and 0 ng of gDNA. The specificity of the amplification as well as possible size differences in the amplicon were checked using a melting curve. Dynein (Esi0298_0008 = LQ0AAB30YA12FM1) and R26S (Esi0072_0068 = CL461Contig1) were selected as reference genes because of their high degree of conservation in our study (log2-ratio < 0.2 in all *E. siliculosus *strains).

### Data deposition

CGH-data (raw and normalized) for strains 2 to 5 were deposited in the ArrayExpress database under accession number ArrayExpress: E-TABM-766. The reference-reference hybridization is available under accession ArrayExpress: E-TABM-967.

## Abbreviations

CDS: coding sequence; CGH: comparative genome hybridization; EST: expressed sequence tag; EsV-1: *Ectocarpus siliculosus *virus 1; FCP: fucoxanthin a/c chlorophyll binding protein; FDR: false discovery rate; gDNA: genomic DNA; GO: gene ontology; GST: glutathione S-transferase; ITS1: internal transcribed spacer 1; TE: transposable element; UTR: untranslated region

## Authors' contributions

TT and SMD conceived the study, together with CB and JYC. CP, SMD, TT, and SR performed the lab-work, and SMD and CP analyzed the results. SMD drafted the manuscript together with TT, JMC, CB, and AFP. All authors approved the final manuscript.

## Supplementary Material

Additional file 1 **List of probes with absolute log2-ratios > 0.5 and > 1 for all examined strains of *E. siliculosus***. Click here for file

Additional file 2 **List of EST derived sequences (singletons and contigs) used for the analysis of FCPs, GSTs, TEs, as well as the corresponding gene models in the *Ectocarpus *genome (for FCPs and GSTs)**. Click here for file

Additional file 3 **".pos" file generated for the *E. siliculosus *gene expression array version 1**. Click here for file
